# Loss of MAPK8IP3 Affects Endocytosis in Neurons

**DOI:** 10.3389/fncel.2022.828071

**Published:** 2022-05-27

**Authors:** Amanda M. Snead, Swetha Gowrishankar

**Affiliations:** Department of Anatomy and Cell Biology, College of Medicine, University of Illinois at Chicago, Chicago, IL, United States

**Keywords:** MAPK8IP3, JIP3, lysosome, DQ-Red BSA, iPSC, neuron, endocytosis, neurodevelopment

## Abstract

Perturbations in endo-lysosomal trafficking pathways are linked to many neurodevelopmental and neurodegenerative diseases. Of relevance to our current study, MAPK8IP3/JIP3, a brain enriched putative adaptor between lysosomes and motors has been previously implicated as a key regulator of axonal lysosome transport. Since *de novo* variants in MAPK8IP3 have recently been linked to a neurodevelopmental disorder with intellectual disability, there is a need to better understand the functioning of this protein in human neurons. To this end, using induced neurons (i^3^Neurons) derived from human iPSCs lacking MAPK8IP3, we demonstrate that loss of hMAPK8IP3 affects endocytic uptake in neurons but does not affect the proteolytic activity of lysosomes in neuronal cell bodies. Our findings indicate that MAPK8IP3 may be a regulator of bulk endocytosis in neurons and that altered endocytic uptake may play a role in MAPK8IP3-linked neurodevelopmental disorders.

## Introduction

*De novo* variants in MAPK8IP3, a putative adaptor protein believed to link cargo to dynein and kinesin motors (Cavalli et al., 2005; Drerup and Nechiporuk, 2013; Gowrishankar et al., 2017, 2021; Cockburn et al., 2018), have been found in children with neurodevelopmental disorders. In recent years, two independent studies identified *de novo* variants in MAPK8IP3 in individuals presenting with intellectual disability as well as brain anomalies including perisylvian polymicrogyria, cerebral or cerebellar atrophy, and hypoplasia of the corpus callosum (Iwasawa et al., 2019; Platzer et al., 2019). Our understanding of MAPK8IP3 function comes largely from studies of its orthologs in *Drosophila melanogaster, Caenorhabditis elegans*, and *Danio rerio* as well as from *Mus musculus* (Drerup and Nechiporuk, 2013; Edwards et al., 2013; Gowrishankar et al., 2017, 2021). Originally identified as Sunday Driver in *D. melanogaster*, studies of MAPK8IP3 and its orthologs (UNC 16 in *C. elegans* and JIP3 in *D. rerio* and *M. musculus*), demonstrate that loss of this protein results in altered axonal lysosome abundance in all these models (Drerup and Nechiporuk, 2013; Edwards et al., 2013; Gowrishankar et al., 2017, 2021). While the *C. elegans* studies proposed a “gate keeper” function for UNC-16 by acting at the axon initial segment to prevent the entry of lysosomes from the soma into axons (Edwards et al., 2013), the studies in *D. rerio* and the mammalian model systems suggest a role for removing axonal lysosomes *via* dynein-based retrograde transport (Drerup and Nechiporuk, 2013; Gowrishankar et al., 2017, 2021). Further supporting a role in retrograde axonal transport, Sunday Driver/ JIP3 was characterized as an adaptor that associates with dynein/dynactin during the transport of axonal injury signals in neurons (Cavalli et al., 2005). JIP3 has since been demonstrated to bind kinesin-1 in addition to the dynein-dynactin complex (Cavalli et al., 2005; Cockburn et al., 2018; Vilela et al., 2019) and TrkB (Huang et al., 2011). Lastly, JIP3 has also been shown to localize to autophagosomes in axons even though it only regulates the transport of mature autolysosomes in these axons (Cason et al., 2021), consistent with previous findings (Drerup and Nechiporuk, 2013; Edwards et al., 2013; Gowrishankar et al., 2017, 2021).

The study by Platzer and colleagues that identified 13 *de novo* variants in *MAPK8IP3* through exome sequencing of 27,232 individuals (majority of whom had been diagnosed with a neurodevelopmental disorder), also examined the effect of some of these variants on axonal lysosome abundance in *C. elegans* (Platzer et al., 2019). Through CRISPR genome editing they engineered six of these variants into the *C. elegans* model and found that only two of those variants exhibited axonal lysosome accumulation like the null version, while multiple variants affected locomotion. This raises the possibility that MAPK8IP3 affects other cellular processes in neurons, which may be adversely affected by the variants linked to neurodevelopmental disease. In this context, we examined whether MAPK8IP3 regulates the degradative capacity of neuronal lysosomes. This is important given that lysosomes are the primary degradative organelles in cells whose functioning is particularly critical for protecting long-lived, post-mitotic cells such as neurons (Ballabio and Bonifacino, 2019; Ferguson, 2019; Gowrishankar et al., 2020) from damaged organelles and misfolded, aggregated proteins. We carried out a lysosomal degradation assay using DQ-Red BSA (dye-quenched Bovine Serum Albumin), a cargo that fluoresces when proteolytically cleaved, in induced neurons (i^3^Neurons) derived from iPSCs lacking MAPK8IP3 (Gowrishankar et al., 2021). While MAPK8IP3 KO i^3^Neurons did not exhibit a decrease in lysosomal proteolytic activity (as measured by DQ-Red BSA fluorescence normalized to Alexa 488-BSA fluorescence in each cell), they did show defects in endocytic uptake of BSA and Dextran, cargoes that serve as markers for fluid phase endocytosis. The finding that loss of MAPK8IP3 does not adversely affect the proteolytic activity of lysosomes in neuronal cell bodies is as important as its potential role in regulating fluid-phase endocytosis. Given variants of MAPK8IP3 are linked to a neurodevelopmental disorder, identifying more neuronal cellular processes that are regulated by MAPK8IP3 will help us understand the neurodevelopmental pathology associated with the different variants.

## Materials and Methods

### MAPK8IP3 KO i^3^Neuron Culture

The JIP3 KO/MAPK8IP3 KO iPSC line was previously generated using CRISPR-Cas9 gene editing (Gowrishankar et al., 2021). iPSCs were maintained in E8 media (Life Technologies) and passaged using accutase (Corning). The iPSCs possess a doxycycline inducible Neurogenin 2 transgene incorporated into the AAVS1 safe harbor locus (Wang et al., 2017), allowing for dependable differentiation of iPSCs into a neuronal fate. These i^3^Neurons were differentiated from iPSCs as described previously (Wang et al., 2017; Fernandopulle et al., 2018; Gowrishankar et al., 2021). MAPK8IP3 KO cells stably expressing LAMP1-GFP were generated previously (Gowrishankar et al., 2021). i^3^Neurons were plated at a density of 30,000 cells per 35 mm glass-bottom dishes (MatTek Life Sciences) coated with 0.1 mg/ml poly-L-ornithine (Sigma Aldrich) and 10 μg/ml mouse Laminin (Gibco). i^3^Neurons were differentiated for 14 days in Cortical Neuron Culture Medium containing KO DMEM F12 (Gibco) B27 supplement (Thermo Fisher), 10 ng/ml BDNF and NT3 (PeproTech), and 1 μg/ml mouse Laminin. i^3^Neurons were supplemented with Cortical Neuronal Culture Medium every 3–4 days during differentiation.

### Measurement of Lysosomal Proteolytic Activity Using DQ-Red BSA

Lysosomal proteolytic activity was measured using the DQ-Red BSA assay as previously described (Marwaha and Sharma, 2017; Kulkarni et al., 2021; Majumder et al., 2021), with modifications for use in i^3^Neurons. DIV 14 i^3^Neurons were pulsed with equal concentrations of the DQ-Red BSA and Alexa 488-BSA probes (25 μg/ml, Thermo Fischer) for a period of either 5–7 h or 2 h, followed by gentle washes [two times with warm imaging media (IM)]. They were then imaged live in IM at 37°C using a Zeiss 880 confocal microscope in Airyscan mode with a 60× objective (NA 1.4). Healthy i^3^Neurons were identified using brightfield mode and imaged using the 561 and 488 lasers to capture fluorescence of DQ-Red BSA and BSA-488. Fluorescence intensity for each of the two channels was measured following outlining of individual cells using Image J software and the normalized ratio of DQ-Red BSA to BSA-488 intensity was computed for each cell (Supplementary Figure 2). The mean of these per cell ratios was then computed and normalized to the value of Control i^3^Neurons to compare across multiple experiments.

Making of DQ-Red BSA and BSA-488 probe mixture: 10% Cortical Neuron Culture Medium (Gowrishankar et al., 2021) in KO DMEM F12 was equilibrated at 37°C and 5% CO_2_. Probes were added to the 10% Cortical Neuronal Culture Medium and incubated at 37°C and 5% CO_2_ for 5 min for equilibration.

Composition of IM: 20 mM HEPES, 5 mM KCL, 1 mM CaCl_2_, 150 mM NaCl, 1 mM MgCl_2_, 1.9 mg/ml glucose, and BSA, pH 7.4.

### Measurement of Endocytic Uptake of BSA

From data collected as described above, the population mean of BSA-488 and DQ-Red BSA fluorescence intensity were each normalized to the value of Control i^3^Neurons of the same experiment to compare across multiple experiments.

### Measurement of Alexa-647 Dextran Uptake

Prior to the overnight pulse of 10 μg/ml Alexa-647 Dextran (Invitrogen) to label endo-lysosomes (Angarola and Ferguson, 2019) in 10% CM, 2 ml of culture media was saved at 37°C and 5% CO_2_. One hour prior to imaging i^3^Neurons were washed with warm PBS and the saved culture medium was replaced for a One hour washout. Cells were then imaged in IM at 37°C as described above using the 633 laser. Fluorescence intensity was measured for each cell using ImageJ as described above and population means were compared between genotypes.

### Validating Proteolytic Lysosomal Identity of DQ-Red BSA Positive Vesicles

To validate the DQ-RedBSA assay (and confirm that the DQ-Red BSA signal corresponds. To proteolytic activity), avacuolar ATPase inhibitor, Bafilomycin A (Sigma Aldrich; known to inhibit the function of lysosomes), was used (Yamamoto et al., 1998). The DQ-RedBSA assay was performed in MAPK8IP3KOLAMP1-GFP i^3^Neurons with a 5 h pulse with the addition of Bafilomycin A at 100 nM final concentration 1 h into the 5 h pulse. Cells were identified during live imaging using the LAMP1-GFP signal.

### Statistical Analysis

Data represent mean ± SEM unless otherwise specified. All statistical tests were performed using Graphpad Prism9 software. Statistical tests performed are indicated in their respective.

Figure legends [including the number of experiments (N), number of cells (n), statistical test used, and *p* values].

## Results

### Loss of MAPK8IP3 in Neurons Affects Endocytosis but Not Lysosomal Degradation

**Figure 1:**
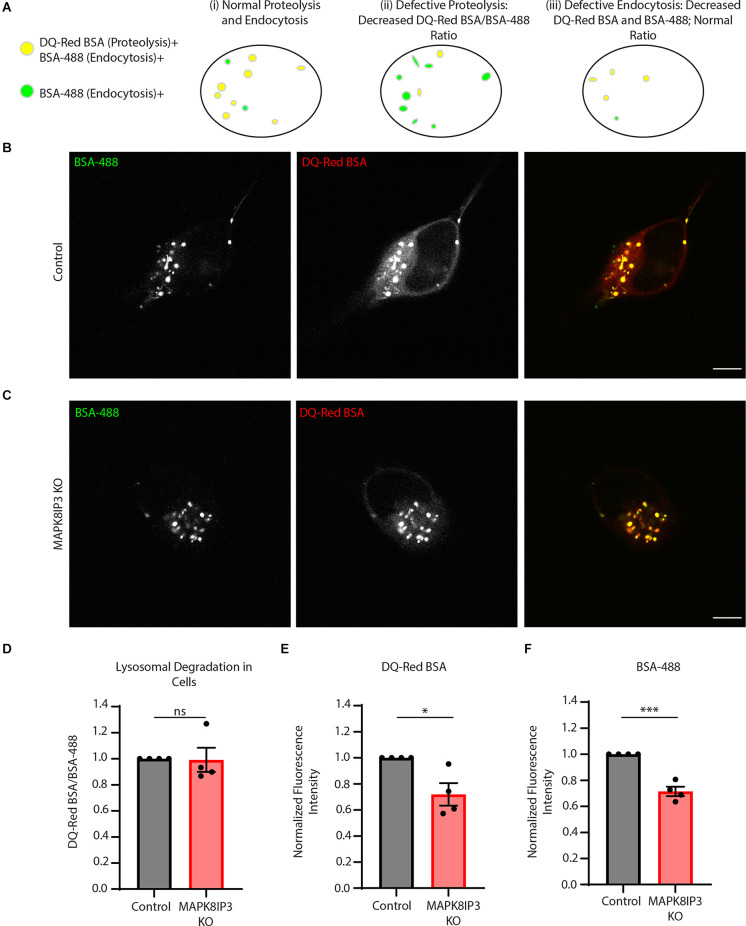
MAPK8IP3 I^3^Neurons show reduced endocytic uptake of BSA. **(A)** Schematic showing that proteolytically active lysosomes are positive for both DQ-Red BSA and BSA-488, shown in yellow, and BSA-488 positive endosomes shown in green (i). If lysosomal proteolysisis perturbed (ii), there will be a decrease in the ratio of DQ-Red BSA fluorescence intensity/BSA-488fluorescence intensity (as both probes are taken up but due to lack of optimal proteolysis, there isreduced DQ-Red BSA fluorescence signal). Lastly, if there is reduced endocytic uptake of both probes, the ratio of DQ-Red BSA fluorescence intensity/BSA-488 fluorescence intensity appears comparableto that of normal cells (indicating normal lysosomal proteolysis) but overall intensity of each of those probes is lower than normal cells (iii). **(B,C)** Representative images of Control and MAPK8IP3 KOi^3^Neurons at DIV14 after 5–7-h incubation with both probes. Scale bar: 5 μm. **(D)** Quantificationshowing the normalized mean DQ-Red BSA/BSA-488 ratio in Control and MAPK8IP3 KO i^3^Neurons from four independent experiments. **(E)** Quantification of DQ-Red BSA fluorescence intensity in Controland MAPK8IP3 KO i^3^Neurons. **(F)** Quantification of BSA-488 fluorescence intensity in Control andMAPK8IP3 KO i^3^Neurons. **(D–F)** Quantifications represent mean ± SEM across four independentexperiments (*P < 0.05, ***P < 0.001; ns, not significant; unpaired *t*-test). Control = 85 cells; MAPK8IP3 KO = 96 cells.

Our assay examining lysosomal proteolytic activity in Control and MAPK8IP3 KO i^3^Neurons, as read out by fluorescence of DQ-Red BSA normalized to fluorescence of BSA-488 for each cell, revealed that the MAPK8IP3 i^3^Neurons do not have reduced lysosomal proteolytic activity compared to Control i^3^Neurons (Figures 1A–D). However, it was apparent from the images (Figures 1B,C) that the fluorescence signal from both channels was dimmer in MAPK8IP3 KO i^3^Neurons compared to Control i^3^Neurons. Indeed, our analysis indicated that the fluorescence intensity of each of the BSA probes was reduced by 28% in MAPK8IP3 KO i^3^Neurons compared to Control (Figures 1E,F). While a drop in DQ-Red BSA in MAPK8IP3 KO neurons alone with BSA-488 fluorescence remaining comparable would indicate a proteolysis defect, a drop in fluorescence intensity of both probes is suggestive of defective endocytic uptake of these probes (Figure 1A). The drop in fluorescence intensity could also result from reduced axonal transport to the soma of BSA-loaded endocytic vesicles, given the known role of MAPK8IP3 in regulating axonal endo-lysosome transport (Gowrishankar et al., 2021). While the KO i^3^Neurons did not exhibit a defect in lysosomal proteolysis, we confirmed that they exhibited the previously demonstrated phenotypes (Gowrishankar et al., 2021) of axonal lysosome accumulation (Supplementary Figure 1).

**Figure 2:**
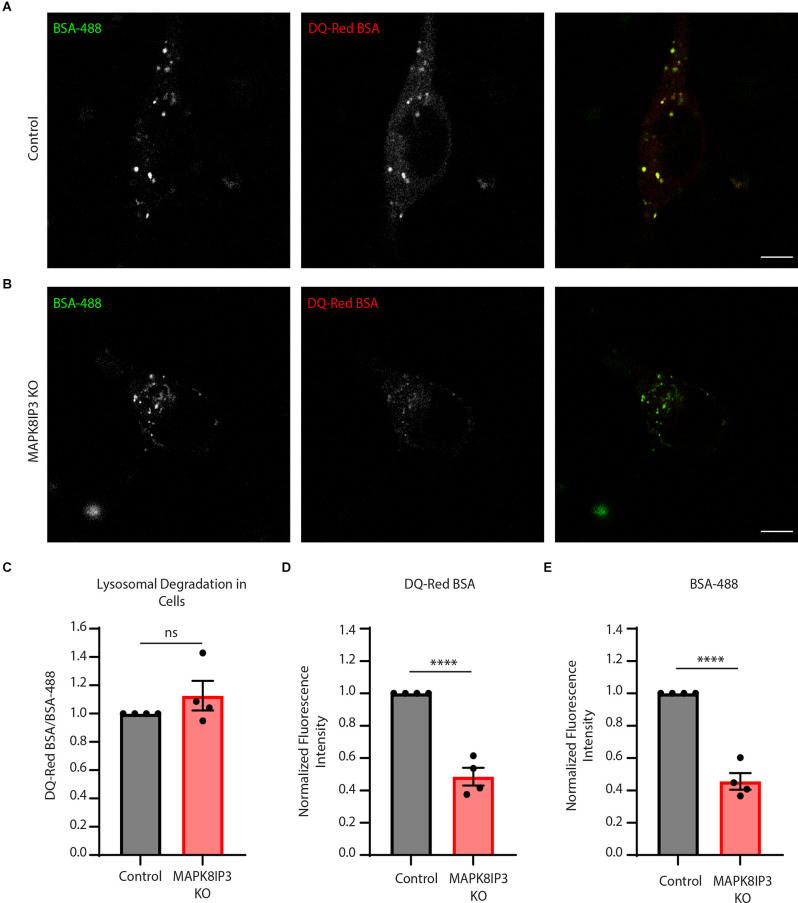
MAPK8IP3 KO i^3^Neuron endocytic uptake is more dramatically decreased with a shorter pulse.**(A,B)** Representative images of Control and MAPK8IP3 KO i^3^Neurons at DIV14 after 2-h pulse withDQ-Red BSA and BSA-488, sclae bar: 5 μm. **(C)** Quantification of mean normalized DQ-Red BSA/BSA-488 mean ratios in Control and MAPK8IP3 KO i^3^Neurons. **(D)** Quantification of DQ-Red BSAfluorescence intensity normalized to Control. **(E)** Quantification of BSA-488 fluorescence intensitynormalized to Control. **(C–E)** Quantifications show mean ± SEM for two independent experiments,each with two technical repeats (****P < 0.0001; ns, not significant; unpaired *t*-test). Control = 75; MAPK8IP3 KO = 54 cells.

The relatively longer pulses of 5–7 h were carried out to ensure the trafficking of endocytosed probes to lysosomes (Marwaha and Sharma, 2017; Kulkarni et al., 2021). Since our findings suggested a potential endocytic defect (Figures 1A,E,F), we next examined the difference in fluorescence following a shorter pulse of 2 h. This is because, with longer pulses, other cellular trafficking events such as recycling or regurgitation (Mayor and Pagano, 2007; Kumari et al., 2010) may also play a role and obscure the extent of the endocytic defect. Indeed, we found a much more dramatic and significant decrease in the signal of both probes in MAPK8IP3 KO i^3^Neurons at 2 h (Figures 2A–E).

### Loss of MAPK8IP3 Affects Endocytosis of Fluorescently Tagged Dextran, a Well-Established Reporter of Fluid Uptake in Cells

**Figure 3:**
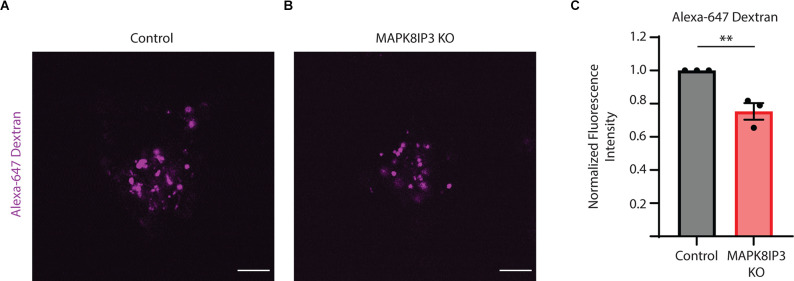
Dextran uptake is reduced in MAPK8IP3 KO i^3^Neurons. **(A,B)** Representative images of Control and MAPK8IP3 KO i^3^Neurons at DIV15 after overnight Alexa-647 Dextran incubation and 1 h washout. Scale bar: 5 μm. **(C)** Quantification of Alexa-647 Dextranfluorescence intensity normalized to Control. Mean ± SEM from three independent experiments (**P < 0.01;unpaired *t*-test). Control = 60 cells; MAPK8IP3 KO = 74 cells.

Given the strong decrease in BSA uptake in MAPK8IP3 KO i^3^Neurons, we examined how the uptake of fluorescently tagged Dextran (Alexa-647 Dextran) was altered in these MAPK8IP3 KO i^3^Neurons compared to Control i^3^Neurons. We found a similar decrease in Alexa-647 Dextranfluorescence in MAPK8IP3 KO i^3^Neurons compared to Control i^3^Neurons (25% reduction, Figures 3A–C) as we did with BSA (28% reduction, Figures 1E,F). Collectively, these results suggest that while loss of MAPK8IP3 does not reduce the proteolytic activity of lysosomes in the neuronal cell body, it does affect endocytic uptake of certain cargo in these human i^3^Neurons.

## Discussion

In this study, we use i^3^Neurons that lack MAPK8IP3 (Gowrishankar et al., 2021) to identify defects in endo-lysosomal traffic that arise from the loss of this protein. Given two independent and recent studies have implicated *de novo* variants in *MAPK8IP3* as a cause of a neurodevelopmental disease with intellectual disability and variable brain anomalies (Iwasawa et al., 2019; Platzer et al., 2019), identifying neuronal cellular processes altered by changes to MAPK8IP3 is critical. Our experiments using MAPK8IP3KO i^3^Neurons reveal a potential new role for the MAPK8IP3 protein in regulating endocytic uptake in neurons, in addition to its well-established role in regulating axonal transport (Cavalli et al., 2005; Huang et al., 2011; Drerup and Nechiporuk, 2013; Gowrishankar et al., 2017, 2021; Cockburn et al., 2018). Of equal importance, our studies suggest that loss of MAPK8IP3 does not affect the proteolytic activity of lysosomes in the neuronal cell body. A critical aspect to understanding how the different *MAPK8IP3* variants contribute to neurodevelopmental pathology will involve examining how these variants affect processes such as endocytosis, axonal lysosome abundance, and lysosome function when compared to complete loss of the protein. Such studies would help determine if these variants (several of which are missense mutations) represent loss of function mutations. For instance, the study by Platzer and colleagues which used the CRISPR-Cas9 system to target six conserved positions in *C. elegans*, found that two of the six human alterations resulted in elevated axonal lysosome density while five affected locomotion (Platzer et al., 2019).

The endocytic uptake of Dextran (10 kDa) into cells normally occurs *via* a clathrin and dynamin independent endocytic pathway, as well as by macropinocytosis, a form of bulk endocytosis that relies on actin polymerization to form around extracellular material (Mayor and Pagano, 2007; Grant and Donaldson, 2009; Kumari et al., 2010; Lin et al., 2020). Interestingly, MAPK8IP3/JIP3 has been implicated in regulating macropinocytosis (Williamson and Donaldson, 2019) as well as fast endosomal recycling and endosome movement in cultured cell lines, through its interaction with the small GTPase ARF6 (Isabet et al., 2009; Marchesin et al., 2015). JIP3, post-recruitment *via* ARF6, is proposed to aid in the completion of macropinosome formation through a coupling of the dynein motor to the growing structure in HT1080 cells, which exhibit constitutive macropinocytosis on the expression of an active form of H-Ras (Williamson and Donaldson, 2019). Interestingly, some of the *MAPK8IP3* variants linked with neurodevelopmental pathology exhibit mutations in the leucine zipper domain (Platzer et al., 2019), which is implicated in ARF6 interaction (Isabet et al., 2009). However, the role of MAPK8IP3 in regulating macropinocytosis in neurons has not been demonstrated thus far. Our results showing reduced Dextran and BSA uptake in MAPK8IP3 KO i^3^Neurons could suggest that MAPK8IP3 plays a similar role in regulating macropinocytosis as observed previously in cultured cancer lines (Marchesin et al., 2015). While Dextran can enter the cell through multiple endocytic pathways, including constitutive clathrin-independent routes and macropinocytosis (which is usually stimulated), we propose that MAPK8IP3 affects macropinocytosis in human neurons, based on its previously demonstrated role in this process in HT1080 cells (Williamson and Donaldson, 2019). This could explain the modest but consistent drop of 25% in Dextran uptake in the KO i^3^Neurons, as other redundant pathways for Dextran entry may be unaffected. Some of the reduction in fluorescence intensity of the endocytic cargo (Dextran and/or BSA) could also result from reduced axonal transport to the soma of cargo-loaded endocytic vesicles. However, similar BSA uptake experiments in the hippocampal neurons suggest that the number of BSA-loaded vesicles in axons is about ten percent of those in dendrites (Kulkarni et al., 2021), which in turn is less than those in soma, suggesting that a block in axonal transport of endocytosed vesicles could only partially account for decreased fluorescence intensity. In further support of a link between MAPK8IP3 and macropinocytosis, loss of MAPK8IP3 in i^3^Neurons causes focal accumulation of actin and Myosin II (Rafiq et al., 2022), both of which are involved in macropinosome formation (Araki et al., 2003; Jiang et al., 2010; Williamson and Donaldson, 2019; Lin et al., 2020). Future studies that include the use of inhibitors such as amiloride (Koivusalo et al., 2010; Yerbury, 2016; Aravamudhan et al., 2020; Ueda et al., 2021) and blebbistatin (Kolpak et al., 2009; Williamson and Donaldson, 2019) in these uptake assays will shed light on whether MAPK8IP3 is involved in macropinocytosis in neurons. The extent and nature of stimuli that induce macropinocytosis in neurons is not as extensively characterized (Lin et al., 2020). However, macropinocytosis has been identified as a means for viral entry into neurons (Kalia et al., 2013; Aravamudhan et al., 2020), propagation of protein aggregates between neurons in culture (Münch et al., 2011; Holmes et al., 2013), and as a mediator of the axon growth cone turning and collapse (Kabayama et al., 2009; Kolpak et al., 2009). Additionally, a role for bulk endocytosis at the synapse is suggested to be a mechanism for maintaining the size of the axon terminal during prolonged activation (Bonanomi et al., 2008). Interestingly, several of the individuals with MAPK8IP3 variants present with thinning or hypoplasia of the corpus callosum (Iwasawa et al., 2019; Platzer et al., 2019). Whether these are due to a lack of normal axonal development or axonal degeneration remains to be investigated.

In conclusion, our studies reveal a role for MAPK8IP3 in regulating endocytic uptake in human neurons. Future work will focus on elucidating other molecular machinery that acts in concert with MAPK8IP3 in this pathway, as well as whether MAPK8IP3 variants linked with intellectual disability affect this pathway in neurons.

## Data Availability Statement

The original results presented in the study are included in the article/Supplementary Material, further inquiries can be directed to the corresponding author.

## Author Contributions

AS performed experiments and analyzed data. AS and SG designed experiments and wrote the manuscript. SG helped with data analysis and supervised the project. All authors contributed to the article and approved the submitted version.

## Conflict of Interest

The authors declare that the research was conducted in the absence of any commercial or financial relationships that could be construed as a potential conflict of interest.

## Publisher’s Note

All claims expressed in this article are solely those of the authors and do not necessarily represent those of their affiliated organizations, or those of the publisher, the editors and the reviewers. Any product that may be evaluated in this article, or claim that may be made by its manufacturer, is not guaranteed or endorsed by the publisher.
