# Supplementary material for: Loss of MAPK8IP3 Affects Endocytosis in Neurons
Source: Front Cell Neurosci. 2022 May 27;16:828071. doi: 10.3389/fncel.2022.828071 (PMC9196590; doi:10.3389/fncel.2022.828071)
Supplement: Supplementary file 1 [file Presentation_1.pdf]

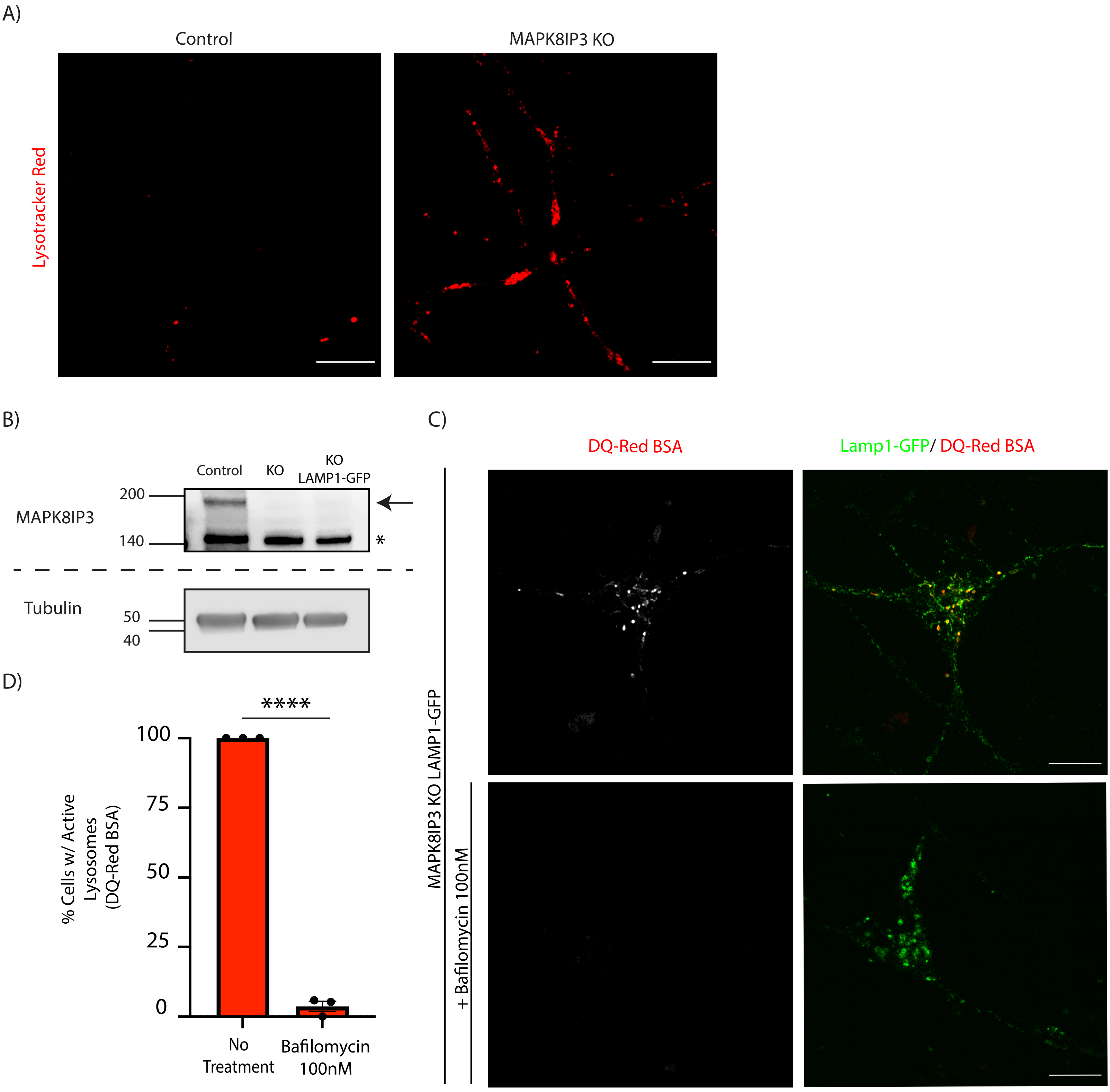

Supplemental Figure 1

Supplemental Figure 1: Validation of axonal phenotypes of MAPK8IP3 KO i³Neurons

A, B) Representative images of cells labeled with lysotracker showing axonal buildup of acidic organelles in the MAPK8IP3 KO i³Neurons while Control i³Neurons have relatively fewer organelles in their axons. C) Western blot showing loss of MAPK8IP3 protein in MAPK8IP3 KO i³Neurons. MAPK8IP3 (arrow) and tubulin (loading control) are probed in Control, MAPK8IP3 KO, and MAPK8IP3 KO LAMP1-GFP DIV21 i3Neurons. Asterisk indicates non-specific band. (D) Quantification of DQ-Red BSA positive lysosomes in MAPK8IP3 KO DIV10 i³Neurons expressing LAMP1-GFP following 5-hour incubation with DQ-Red BSA with or without addition of Bafilomycin A (mean ± SEM from three independent experiments; Control n = 47 cells; Bafilomycin n = 46 cells; ****$P < 0.0001$, unpaired t test). E) Representative images showing DQ-Red BSA positive vesicles and lack of DQ-Red BSA vesicles with Bafilomycin treatment. Scale bar, 10μm.

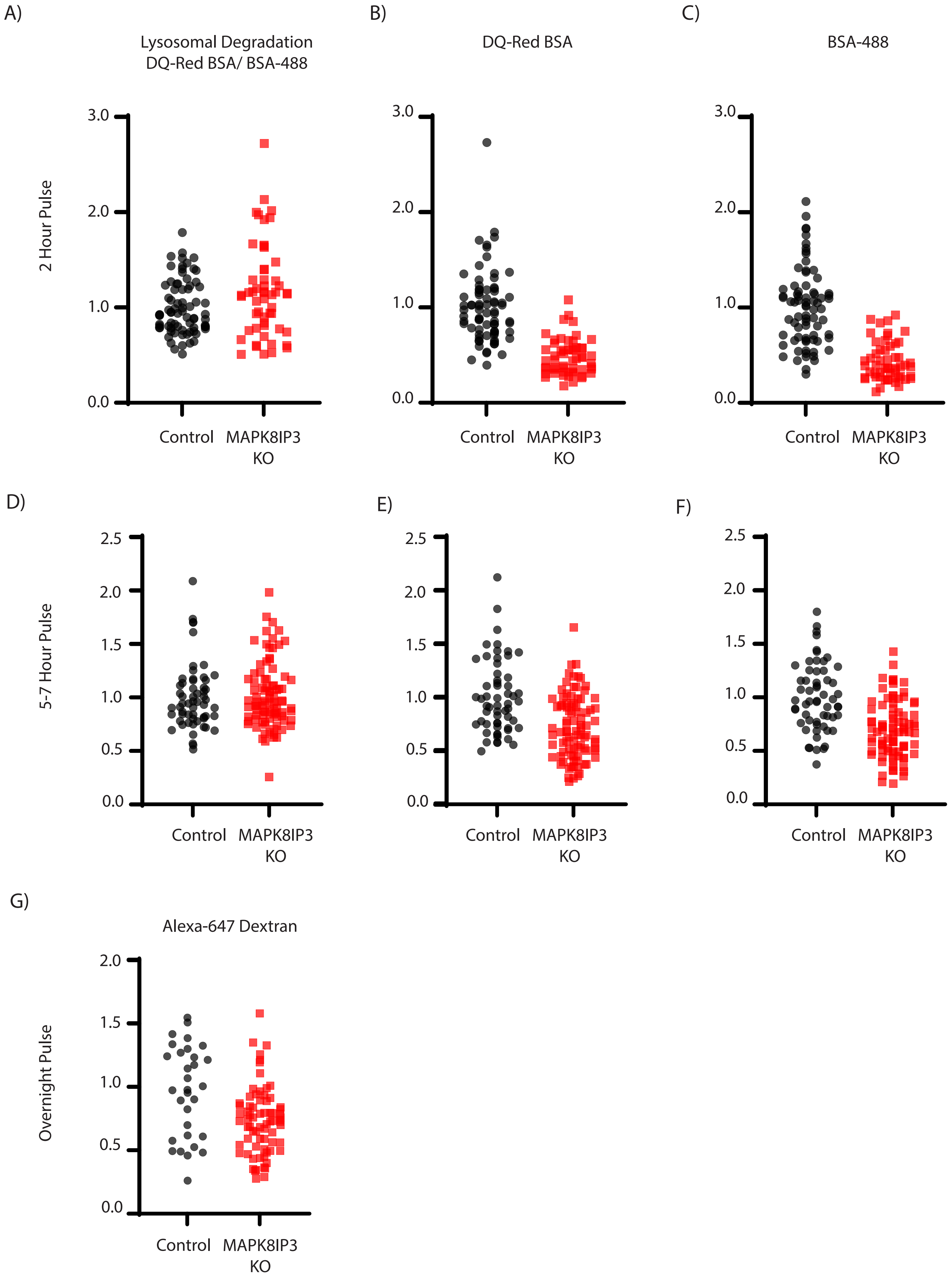

Supplemental Figure 2

Supplemental Figure 2: Depiction of cell-to-cell variability in lysosomal proteolytic and endocytic assays in i^{3}Neurons

Graphs (A, D) depict the lysosomal degradation (DQ-Red BSA/BSA-488 ratio) in each cell across three experiments, where ratio from each cell was in turn normalized to population mean of Control i^{3}Neurons from same experiment. Graphs show DQ-Red BSA (B,E), BSA-488 (C,F) and Dextran (G) uptake in individual cells across experiments normalized to population mean of Control i^{3}Neurons from their respective experiments.
